# Recent Advances in Beta-Alanine Production via Enzymatic Catalysis and Microbial Whole-Cell Catalysis

**DOI:** 10.3390/biology15110885

**Published:** 2026-06-03

**Authors:** Jie Yu, Peikun Ma, Jiabei Zhang, Hongyang Zhang, Hang Tie, Haihua Ruan

**Affiliations:** 1 Dong-E E-Jiao Co., Ltd., Liaocheng 252201, China; yujie1@dongeejiao.com; 2 Tianjin Key Laboratory of Food Biotechnology, School of Biotechnology and Food Science, Tianjin University of Commerce, Tianjin 300134, China; pkd7328@163.com (P.M.); cf04202024@163.com (J.Z.); zhyang@tjcu.edu.cn (H.Z.); 3 Chinese Academy of Inspection & Quarantine Greater Bay Area, Zhongshan 528437, China; 15620971896@126.com

**Keywords:** beta-alanine, biosynthesis, enzymatic catalysis, microbial whole-cell catalysis

## Abstract

Beta-alanine, a naturally occurring non-proteinogenic beta-amino acid, is widely used in delaying fatigue, enhancing exercise performance, and alleviating hyperuricemia. It also serves as a three-carbon platform for the synthesis of various high-value compounds, thus showing extremely broad market prospects. However, the practical application of beta-alanine is severely limited by the harsh reaction conditions and abundant by-products in chemical synthesis, as well as the low endogenous content and complex biosynthetic pathway. Recently, advances have been made in the one-pot, one- or two-step production of beta-alanine using genetically engineered recombinant enzymes, and in the microbial synthesis of beta-alanine via whole-cell biocatalysis. These advances are based on a series of attempts, including the enzymatic conversion to beta-alanine using 1,3-diaminopropane (DAP), L-aspartate (L-Asp), fumarate, or 3-aminopropionitrile as substrates, and the whole-cell biosynthesis of beta-alanine by regulating metabolic flux from carbon sources (e.g., glucose, oil, and glycerol) to L-Asp—the precursor for beta-alanine synthesis catalyzed by L-aspartate-α-decarboxylase (ADC). This study provides a rational theoretical basis and valuable practical references for the future industrial application of beta-alanine.

## Simple Summary

Beta-alanine is a unique natural amino acid that does not build proteins, but it brings plenty of tangible benefits to daily health and physical activity. In addition to its health-related applications, it is widely used in cosmetics and food additives, and also serves as a critical precursor in the chemical, pharmaceutical and material industries. As a versatile three-carbon building block for synthesizing various high-value chemicals, beta-alanine boasts extremely promising market value across multiple sectors. For a long time, the large-scale industrial production of beta-alanine has faced major hurdles. Traditional chemical synthesis generates large amounts of unwanted byproducts and is harmful to the environment, while natural biological production suffers from complex metabolic regulation and low efficiency, making mass manufacturing difficult. This paper summarizes the latest progress in two biosynthesis methods: enzymatic conversion and microbial synthesis of beta-alanine. It explores the theoretical basis for sustainable and green beta-alanine production, which has great potential for further innovations and large-scale industrial applications in the future.

## 1. Introduction

Beta-alanine, a naturally occurring non-proteinogenic beta-amino acid, is reported to be synthesized in animals, plants [1], bacteria [2], and fungi [3]. Until now, beta-alanine has been reported to be widely utilized in pharmaceuticals [4,5], nutraceuticals [6], functional foods [7], and cosmetic products [8]. Beta-alanine is a well-known substrate for carnosine synthetase; its supplementation leads to increased carnosine biosynthesis [5]. Carnosine acts as an effective anti-glycation and anti-lipoxidation agent; its beta-alanine moiety directly scavenges oxidized carbohydrates and lipids, modulates the buffering of hydrogen ions generated during intense exercise, and thereby delays fatigue and enhances exercise performance. A meta-analysis including six databases and a total of 331 pooled participants demonstrated that beta-alanine supplementation for 4 weeks provides the greatest ergogenic benefits, with an effective daily dose ranging from 5.6 to 6.4 g, and is most beneficial for exercise lasting 4–10 min [9]. Beta-alanine acts as a naturally occurring small-molecule neurotransmitter in the central nervous system (CNS). It is released upon electrical stimulation, possesses specific binding sites, and inhibits neuronal excitability [10]. Upon the increased risk of hyperuricemia (HU) and gout associated with high-altitude exposure, beta-alanine intervention can ameliorate hyperuricemia by the comprehensive analysis of the serum metabolome and phenome in both discovery and validation cohorts of Han Chinese individuals [11]. In plants and fungi, beta-alanine plays a key role in the formation of pantothenic acid that is required for post-translational modification of enzymes involved in specialized metabolites (secondary metabolites) biosynthesis including fatty acid synthases, polyketide synthases, non-ribosomal peptide synthetases and other secondary metabolites biosynthetic enzymes [12].

From a structural perspective, beta-alanine serves as a three-carbon platform for the synthesis of various high-value compounds, including carnosine (a functional supplement for cardiometabolic disorders and autism spectrum disorder) [13], pantothenic acid (vitamin B_5_) [14], pantethine (a promising agent for adjuvant therapy) [15], coenzyme A (involved in fatty acid metabolism) [16], and poly-beta-alanine (used in cosmetics and nanocomposite fabrication) [17]. Growing demand for sports nutrition and health supplements is projected to drive the global beta-alanine market [18]. Given the above functional properties, beta-alanine is recognized as a vital intermediate that participates in diverse biological pathways, highlighting a strong demand for efficient, safe, and green production strategies. Nevertheless, traditional chemical approaches for beta-alanine synthesis suffer from harsh reaction conditions, high energy consumption, environmental pollution, and the generation of toxic by-products [19]. In recent years, considerable progress has been achieved in the synthesis of beta-alanine via a biological catalysis process based on its comprehensive advantages of mild reaction conditions, including renewable and low-cost feedstocks, flexibly optimized reactions by modifying key enzymes, enhancing cofactor regeneration, regulating intermediate metabolism and strengthening product export, etc. In this review, we comprehensively summarize recent advances in beta-alanine biosynthetic strategies with an emphasis on dissecting their underlying metabolic pathway. Thereby, we further provide perspectives to inform the rational improvement of beta-alanine production titers.

## 2. Strategies for the Biosynthetic Production of Beta-Alanine

According to Global Info Research’s latest study, the global beta-alanine market size was valued at USD 89.04 million in 2025 and is forecast to reach a readjusted size of USD 121 million by 2032 with a CAGR of 4.5% during the review period [20], which prompts a huge demand for beta-alanine synthesis. At the early stage, chemical synthesis for beta-alanine is the mainstream approach. Until now, acrylic acid [21], acrylonitrile [22], beta-aminoacrylonitrile [23], beta-aminopropanol [24] and succinimide [22] have been reported to be used as substrates for beta-alanine synthesis. These traditional processes from the last century are still predominant, with acrylic acid and acrylonitrile being the most widely used substrates.

Compared with traditional chemical synthesis, biosynthetic methods provide a greener, more efficient and sustainable technical solution for modern industrial manufacturing [25]. Firstly, they are highly environmentally friendly, as most reactions are carried out under mild conditions such as normal temperature and pressure, reducing energy consumption and avoiding the use of toxic and harmful chemical reagents, thus lowering environmental pollution [26]. Secondly, biocatalysts like enzymes possess excellent specificity and selectivity, enabling precise synthesis of target products with fewer by-products and higher product purity, which simplifies subsequent separation and purification processes [27]. In addition, biosynthetic routes can utilize renewable biological feedstocks such as glucose and biomass, thereby enabling sustainable production and reducing reliance on petrochemical resources. Furthermore, with the rapid advancement of synthetic biology, biosynthetic pathways can be flexibly designed and optimized to efficiently synthesize complex natural products, drugs, and fine chemicals that are difficult to achieve through chemical synthesis methods [28]. The aspect of beta-alanine biosynthesis strategies mainly includes enzyme catalysis and the microbial synthesis of beta-alanine via whole-cell biocatalysis.

### 2.1. Enzyme Catalytic Biosynthesis of Beta-Alanine via Cell-Free Biotransformation

Recently, cell-free biotransformation has emerged as a promising alternative to synthesize industrial products [29]. This approach allows the precise and independent regulation of key reaction parameters, including pH, temperature, and cofactor concentrations, thereby enabling direct optimization of enzyme stoichiometry and catalytic activity [30]. By eliminating cell membrane barriers and intracellular metabolic interference, cell-free systems minimize undesired side reactions and by-product formation, thus enhancing both reaction specificity and conversion efficiency [31]. Furthermore, this strategy circumvents limitations associated with cell viability and toxicity tolerance, rendering it particularly suitable for the production of compounds that otherwise inhibit cell growth.

**Figure 1:**
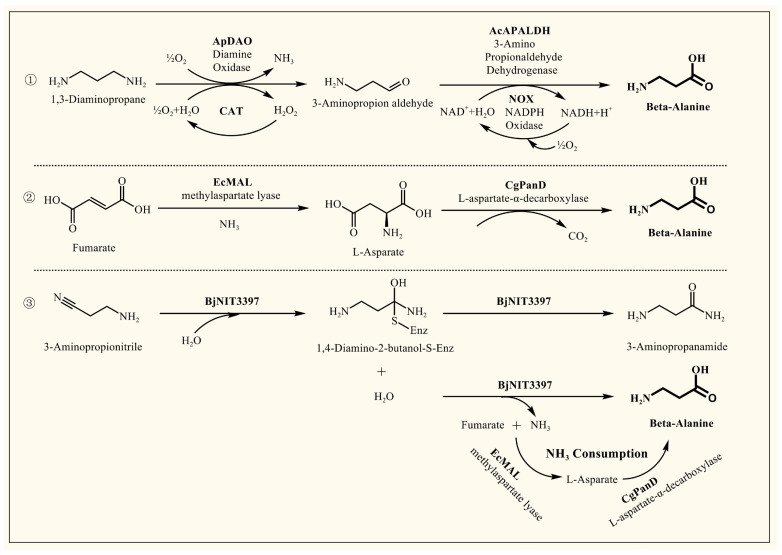
Schematic diagram of enzyme-catalytic biosynthesis of beta-alanine from: ➀ Two-enzyme cascade for beta-alanine synthesis starting from DAP in a cell-free enzymatic biotransformation via 3-aminopropion aldehyde (3-APAL) catalyzed by two primary enzymes, *A. pascens* diamine oxidase (ApDAO) and *A. crystallopoietes* 3-APAL dehydrogenase (AcAPALDH) and two auxiliary enzymes, NOX and catalase; ➁ two-enzyme cascade for beta-alanine synthesis starting from fumarate in a cell-free enzymatic biotransformation via L-Asp catalyzed by two enzymes including *E. coli* methylaspartate lyase (EcMAL) and *C. glutamicum* L-aspartate-α-decarboxylase (CgPanD); ➂ one step conversion of 3-aminopropionitrile to beta-alanine via nitrilase in *E. coli* whole cell lysate, and two auxiliary enzymes, EcMAL and CgPanD, for NH_3_ consumption, L-Asp co-production and by-product 3-aminopropanamide elimination.

In cell-free biotransformation biosynthesis of beta-alanine, the core optimization objectives for high product yield encompass reducing reaction steps, adopting stable and readily available key enzymes, lowering coenzyme consumption, achieving efficient coenzyme regeneration, and eliminating by-products in a timely manner to shift the reaction equilibrium toward target product formation. By summarizing the research about biosynthesis of beta-alanine via cell-free biotransformation, there are four kinds of substrates, including 1,3-diaminopropane (DAP) [18], fumarate [32], L-asparate (L-Asp) [33], and 3-aminopropionitrile [34], that have been reported until now, which have the capability to produce beta-alanine as illustrated in Figure 1.

#### 2.1.1. One-Pot Cell-Free Biosynthesis of Beta-Alanine from DAP via a Two-Step Enzymatic Cascade

In 2025, Shanmugasundaram et al. established a one-pot cell-free biotransformation system for beta-alanine synthesis from DAP via a novel two-step enzymatic route [18], which converts the three-carbon amine substrate DAP into beta-alanine (Figure 1, Reaction ➀). In the first step, DAP is oxidized to 3-aminopropionaldehyde (3-APAL) by diamine oxidase (DAO). DAO uses oxygen as an electron acceptor, generating H_2_O_2_ and NH_3_ as by-products. The DAO employed primarily in this study was evolved from commercially available porcine kidney DAO, which was then found to exhibit obviously lower specific activity than the *Arthrobacter pascens* (*A. pascens*) DAO (ApDAO), which was finally found to display a higher catalytic efficiency with a *k*_cat_/*K*_m_ value of 1.4 × 10^6^ M^−1^s^−1^. In the second step, the intermediate 3-APAL is further oxidized to beta-alanine by 3-APAL dehydrogenase (AcAPALDH) from *Arthrobacter crystallopoietes* (*A. crystallopoietes*) [35], which has a *K*_m_ value of 0.003 mM and shows a high affinity to 3-APAL. This reaction depends on NAD^+^ as a cofactor, producing NADH and H^+^ [36]. This work presents two notable advantages worthy of reference. First, the introduction of catalase (from bovine liver, *K*_m_ = 0.35 mM) prevents oxidative inactivation of enzymes by rapidly scavenging H_2_O_2_ generated during DAO catalysis. Second, an efficient NAD^+^/NADH recycling system is established by introducing NADH oxidase (NOX) from *Lactococcus lactis* (*K*_m_ = 0.004 mM), which regenerates NAD^+^ from NADH [37]. This exogeneous NAD^+^ regeneration system was chosen for its near irreversibility, non-toxicity, low cost, use of benign substrates, and formation of harmless by-products. Under optimized conditions (pH 9, 37 °C), fed-batch enzymatic biotransformation using ApDAO successfully produced 1.1 g/L beta-alanine within 4 h as shown in Table 1 [18]. This system demonstrates high potential for industrial-scale production and serves as a robust chassis for the synthesis of other high-value compounds via beta-alanine, including carnosine, pantothenic acid, and pantethine.

#### 2.1.2. One-Pot Cell-Free Dual-Enzyme Cascade System for Beta-Alanine Synthesis from Fumarate

Beyond DAP, the direct decarboxylation of L-Asp to beta-alanine—catalyzed by L-aspartate-α-decarboxylase (ADC)—represents a more ideal biosynthetic route as illustrated by the second step of Reaction ➁ (Figure 1). In 2014, Shen et al. found that a cloned and expressed CgPanD from *Corynebacterium glutamicum* (*C. glutamicum*) encoding ADC catalyzes a-decarboxylation of L-Asp to beta-alanine [33]. Further investigation showed that the purified CgADC was optimal at 55 °C and pH 6 with excellent stability at 16–37 °C and pH 4–7. A pH-stat-directed, fed-batch feeding strategy was developed for the enzymatic synthesis of beta-alanine to keep the pH value within 6–7.2 and thus attenuate substrate inhibition. A maximum conversion of 97.2% was obtained with an initial 5 g /L L-Asp. The final beta-alanine concentration was 12.85 g/L after 36 h of reaction as shown in Table 1. This is the first study concerning the enzymatic production of beta-alanine by using ADC. The enzymatic production of beta-alanine has seen significant advancements with the expansion of protein engineering techniques. However, this ADC-catalyzed beta-alanine synthesis is generally accomplished via whole-cell enzymatic catalysis [38] instead of enzyme conversion systems in vitro due to the high cost of the substrate L-Asp and the substrate inhibition, limiting the industrialization of this method [39]. On the other hand, a major challenge remains that the genetically modified ADC obtained via protein engineering tends to easily lose its activity and is difficult to be stably supported by its coenzyme (e.g., pyridoxal 5′-phosphate) when functioning as a catalyst [40].

As an alternative, Ni et al. took advantage of the significantly lower cost of fumarate compared to L-aspartate (L-Asp) to develop a cell-free dual-enzyme cascade system for beta-alanine synthesis [32]. This system employs recombinant *Escherichia coli* (*E. coli*) O157:H7 methylaspartate lyase (EcMAL) and *C. glutamicum* aspartate decarboxylase (CgPanD), both of which were heterologously expressed and purified from engineered *E. coli*. In this cascade, EcMAL first catalyzes the stereospecific hydration of fumarate to form L-Asp, as illustrated in Reaction ➁ of Figure 1. Subsequently, CgPanD mediates the α-decarboxylation of L-Asp, yielding beta-alanine with CO_2_ as the sole by-product. Theoretically, this process achieves a 1:1 stoichiometric conversion of fumarate to beta-alanine, and the evolution of CO_2_ simplifies the downstream separation and purification of the product [32].

To further optimize this cascade, the authors performed gene mining, sequence alignment, and enzymatic characterization, selecting EcMAL (*K*ₘ = 6.7 mM, *k*_cat_/*K*ₘ = 13 × 10^3^ M^−1^s^−1^) as the core enzyme and identifying CgPanD as the optimal PanD variant. CgPanD exhibited the highest substrate affinity, with a *K*ₘ of 4.3 mM and a *k*_cat_/*K*ₘ value of 0.65 × 10^3^ M^−1^S^−1^ for L-Asp. Under optimized conditions (35 °C, pH 8.0, and an EcMAL: CgPanD concentration ratio of 1:5), the system showed an achieved 80% of the theoretical yield within 120 min, but the final titer of beta-alanine using fumarate was not measured (Table 1).

This study not only demonstrates a cost-effective strategy for beta-alanine biosynthesis but also lays a solid foundation for its future industrial-scale production. However, besides the precise and independent regulation of parameters and intracellular metabolic interference elimination, this system also presents notable limitations, including the high cost associated with enzyme purification and expensive cofactor supplementation such as pyridoxal-5′-phosphate (PLP). Fumarate is mainly derived from fossil oil, which is not sustainable, and enzyme purification for large-scale production is still expensive [41]. The reaction component regeneration system remains a great obstacle for large-scale industrial applications [42].

#### 2.1.3. Enzyme Catalytic Biosynthesis of Beta-Alanine from 3-Aminopropionitrile

Nitrilase (EC 3.5.5.1), an enzyme of the nitrilase superfamily, catalyzes a series of nitriles into corresponding carboxylic acids with the liberation of ammonia in a one-step reaction [43,44]. It has been reported that beta-alanine was produced from 3-aminopropionitrile by microorganisms, *Alcaligenes* sp. OMT-MY14, *Aminobacter aminobrance* ATCC 23314 [45], *Rhodococcus erythropolis* [46], and *Bradyrhizobium japonicum* (*B. japonicum*) USDA110 [47]. Given the importance of beta-alanine, the nitrilase BjNIT3397 from *B. japonicum* strain USDA110 was examined for the hydrolysis of 3-aminopropionitrile as illustrated in Figure 1 Reaction ➂. It is worth noting that the nitrilase used in this study was prepared from *E. coli* cell lysate, representing another type of cell-free biotransformation system. It has been found that nitrilase BjNIT3397 effectively hydrolyzed 3-aminopropionitrile with substrate concentration up to 3 M (210 g/L) at pH 7.3 and temperature 30 °C. However, with the increase in substrate concentration from 0.6 to 3 M, 3-aminopropanamide was formed and its percentage in the products was increased up to 33% in the product mixture, demolishing the production of beta-alanine. Although the final titer of beta-alanine was not determined, the transformation efficiency was clearly depicted in this research, as the yield of β-alanine reached 80% theoretical yield within 2 h as shown in Table 1. By disclosing the underlying catalytic mechanism of nitrilase, it is known to hydrolyze nitrile to the corresponding amide [48]. These amide compounds might be formed because of the delayed delivery of the second H_2_O [49]. The addition of the first H_2_O to the nitrile group leads to a thiomidate intermediate, which can break down to produce the corresponding amide product [50], causing some nitrilases to have the same character as nitrile hydratase (Figure 1 Reaction 3) [51]. The amide by-products are detected in the hydrolysis of some other nitriles catalyzed by nitrilase BjNIT3397 [52]. In order to reduce the formation of 3-aminopropanamide, substrate (fumarate) and two enzymes (EcMAL and CgPanD) were simultaneously added into the reaction system to consume the byproduct ammonia [34]. As expected, the reaction was shifted toward the robust formation of beta-alanine, resulting in the decrease of 3-aminopropanamide from 33% to 3% as shown in Figure 1 Reaction 3. Therefore, a tandem reaction strategy is developed to effectively prevent the formation of 3-aminopropanamide. This strategy also offers the possibility of producing beta-alanine and L-Asp in one process. This work presents two notable advantages worthy of reference. First, because of its excellent advantages of being easy to acquire, and the enzymatic reaction conditions being mild and requiring no additional coenzymes, the nitrilase-mediated biocatalysis reaction has been continuously arousing interest among scholars with great potential for industrial applications. Second, removal of NH_3_ from the reaction system via EcMAL and CgPanD facilitates the cleavage of the C–N bond, thus shifting the reaction toward the formation of beta-alanine instead of toward the production of byproduct 3-aminopropanamide. Whereas, distinct from the one-pot cell-free enzyme cascades, the synthesis of beta-alanine from 3-aminopropionitrile utilizes the homogenized *E. coli* Rosetta2 (DE3), in which the nitrilase BjNIT3397 was heterologously expressed after induction by IPTG (Isopropyl beta-D-1-thiogalactopyranoside) as the supplier of nitrilase. Simultaneously, *E. coli* K-12 MG1655 harboring EcMAL and CgPanD genes was also used as the enzyme cascade provider responsible for the NH_3_ consumption reaction, facilitating the sustainable production of beta-alanine [47].

In summary, in our viewpoint, as far as the enzymatic conversion for beta-alanine production is concerned, the attempts are still in the early exploratory stage of experimental investigation. From the aspect of techno-economic considerations, the nitrilase-catalyzed one-step reaction from 3-aminopropionitrile showed the greatest potential within these three enzymatic transformations for beta-alanine production, due to the cheap substrate and co-enzyme-free nature in comparison with the expensive fumarate and its pyridoxal-5′-phosphate (PLP), and the ApDAO NAD^+^-NADH regeneration system as a cofactor. A series of limitations needs to be overcome before actual industrial manufacturing. Key aspects are required for continuous conditional optimization in the process of enzymatic conversion to beta-alanine, including the high-throughput screening methods for enzyme variants with high reactivity, excellent thermostability and pH stability; the understanding of the relationship when constructing enzymatic cascade systems that improve reaction efficiency while reducing by-product formation; and the establishment of coenzyme recycling systems, as well as enzyme immobilization and reuse based on the consideration for industrial economic value.

### 2.2. Microbial Synthesis of Beta-Alanine via Whole-Cell Catalysis

Unlike the enzymatic conversion to beta-alanine synthesis in vitro, the microbial production of beta-alanine via whole-cell biocatalysis mainly relies on the host’s native or engineered metabolic pathways, which convert sustainable and eco-friendly biobased feedstocks—primarily sugars and carbohydrates such as glucose, glycerol, and oils—through a specific set of microbial enzymes. Whole-cell systems facilitate efficient multi-step reactions and reduce overall manufacturing costs, as cell lysis and downstream purification processes are unnecessary [53]. Moreover, these whole-cell systems tend to be more sustainable and environmentally friendly, and are able to synthesize regulated, biodegradable by-products [54].

**Figure 2:**
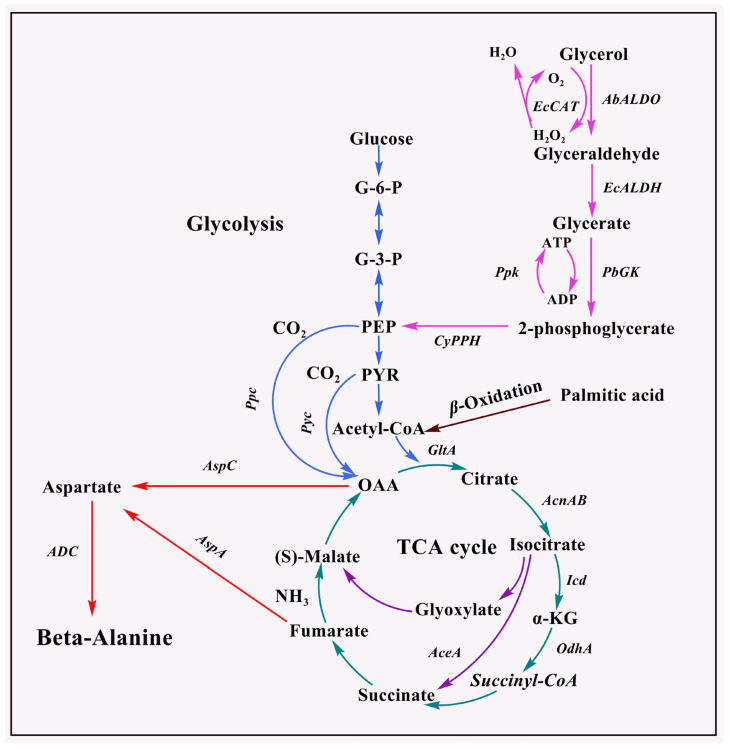
Various whole-cell microbial beta-alanine biosynthesis metabolic pathways with glucose, glycerol and palmitic acid as carbon sources. G-6-P: Glucose 6 phosphate; G-3-P: Glyceralde-hyde-3-phosphate; PEP: phosphoenolpyruvate; PYR: pyruvate; OAA: oxaloacetate; α-KG: α- ke-toglutaric acid; EcCAT: *E. coli* catalase; AbALDO: *A. bacterium* alditol oxidase; EcALDH: *E. coli* lactaldehyde dehydrogenase; Ppk: polyP kinase; PbGK: *P. bacterium* glycerate kinase; CyPPH: *C. ydocella* phosphopy-ruvate hydratase; Ppc: phosphoenolpyruvate carboxylase; Pyc: pyruvate carboxylase; GltA: citrate synthase; AcnAB: aconitase A and B; Icd: isocitrate dehydrogenase; OdhA, α-ketoglutarate dehydrogenase; AceA: isocitrate lyase; AspC: aspartate aminotransferase; AspA: aspartate ammonia-lyase; ADC: L-aspartate-α-decarboxylase.

By summarizing the progress of beta-alanine synthesis via whole-cell catalysis, nearly all studies have used one-step decarboxylation of L-Asp catalyzed by ADC to generate beta-alanine [41], regardless of the microbial strain or the primary carbon sources employed for cell growth as shown in Figure 2. Therefore, recent studies focusing on microbial host engineering for beta-alanine production have mainly centered on redirecting metabolic fluxes and enhancing the decarboxylation of L-Asp. As to the aspect of L-Asp production, it can be acquired from oxaloacetic acid (OAA) or fumarate (Figure 2). Both OAA and fumarate are the core intermediate and indispensable substrates in the tricarboxylic acid (TCA) cycle. Therefore, nearly all the carbon sources and energy suppliers, like glucose, fatty acid, or glycerol, all influence the metabolism of OAA or fumarate, therefore playing regulatory functions on the production of L-Asp and beta-alanine finally.

#### 2.2.1. Microbial Synthesis of Beta-Alanine via Glucose-OAA-L-Asp Pathway

The production of L-Asp and subsequent beta-alanine from glucose as the carbon source mainly relies on the three steps illustrated in Figure 2 by blue arrows. After glucose is absorbed by a bacterium, it is sequentially converted into glucose-6-phosphate (G-6-P), phosphoenolpyruvate (PEP), and pyruvate (PYR) via glycolysis.

First, PEP is catalyzed by phosphoenolpyruvate carboxylase (Ppc), which has long been recognized as the key enzyme responsible for bicarbonate fixation, catalyzing the reaction between CO_2_ and PEP to yield OAA [55]. Subsequently, OAA is then converted to L-Asp by native or heterologously introduced aspartate aminotransferase (AspC) [56]. Therefore, in order to boost metabolic flux toward beta-alanine, the key genes governing precursor accumulation to OAA and L-Asp, like Ppc, are commonly overexpressed to redirect carbon flux through the C4 anaplerotic pathway and promote OAA synthesis [57]. Second, pyruvate can be converted into OAA by pyruvate carboxylase (Pyc), which then feeds into the beta-alanine biosynthetic pathway under the intermediation of L-Asp. Theoretically, overexpression of Pyc facilitates the conversion of pyruvate to L-Asp and beta-alanine, like those of Ppc using PEP as feedstock, as shown in Figure 2 in the blue arrow. Finally, L-Asp is converted into beta-alanine by ADCs (Figure 2, red arrow).

At the early stage of whole-cell synthesis, researchers give more attention to the production of beta-alanine from L-Asp. For instance, an engineered *E. coli* was constructed by heterologously expressing the *C. glutamicum* panD gene (encoding ADC) and overexpressing *E. coli* AspA (encoding aspartate ammonia-lyase) and acs (encoding acetyl-CoA synthetase). As a result, 32.3 g/L of beta-alanine was obtained from fed-batch fermentation [58] under an optimal pH of 6.5 and showed higher activity and thermostability at 37 °C. By establishing an efficient route for L-Asp and beta-alanine synthesis via the PEPC-mediated anaplerotic pathway, Piao et al. achieved production titers of 249 mM (33.1 g/L) for L-Asp and 424 mM (37.7 g/L) for beta-alanine [59]. More recently, heterologous expression of both B. subtilis PanD and *C. glutamicum* AspB (encoding aspartate aminotransferase) in *E. coli* resulted in 85 g/L of beta-alanine from fed-batch fermentation [60]. *C. glutamicum* was also used as a production host, and showed a promising performance, producing 56.5 g/L beta-alanine [61].

**Figure 3:**
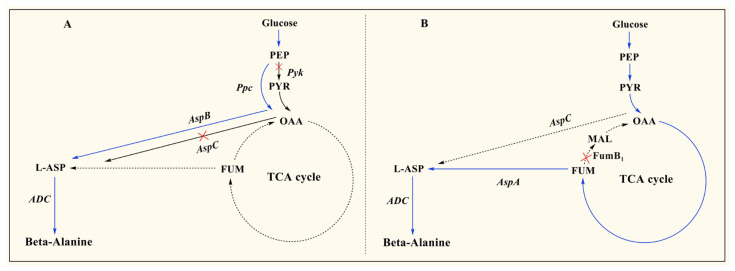
Whole-cell catalysis production of beta-alanine in *E.coli* via glucose-PEP-OAA pathway by *AspB* and *ADC* (**A**), and glucose-TCA-FUM pathway by *AspA* and *ADC* (**B**). PEP: phosphoenolpyruvate; PYR: pyruvate; OAA: oxaloacetate; FUM: fumarate; *Pyk*: pyruvate carboxykinase; *Ppc*: phosphoenolpyruvate carboxylase; *AspC*: aspartate aminotransferase derived from *E.coli*; AspA: aspartate ammonia-lyase derived from *E. coli*; *AspB*: aspartate aminotransferase derived from *C. glutamicum*.

Besides that, the supply of L-Asp—the direct precursor for beta-alanine synthesis—has been boosted by driving metabolic flux toward its upstream intermediates OAA and/or FUM [62]. Moreover, since phosphoenolpyruvate (PEP) serves as a critical precursor for both OAA and fumarate biosynthesis (Figure 2), elevating PEP pools has become a common strategy to improve beta-alanine titers. Accordingly, Hu et al. designed two distinct metabolic engineering strategies to enhance beta-alanine production [63]. As depicted in Figure 3A, Hu et al. heterologously expressed CgAspB (*C. glutamicum* aspartate aminotransferase). To redirect metabolic flux toward OAA-derived L-Asp synthesis, the native *aspC* gene of *E. coli* was first inactivated to eliminate the interference with *C. glutamicum aspB*-mediated transamination [63]. Additionally, the OAA synthesis pathway was further enhanced by overexpressing *ppc*, which catalyzes the carboxylation of PEP to OAA. Consequently, the beta-alanine titer reached 1.00 g/L, a value significantly higher than the 0.62 g/L achieved via the FUM-dependent pathway [62]. 

To date, nevertheless, these yields still fall short of the theoretical maximum. This is primarily attributed to the diversion of a portion of metabolic flux into the TCA cycle, which is essential for energy generation to maintain cellular functions. Furthermore, disruption of the glycolytic pathway in engineered strains to maximize flux toward OAA often leads to impaired cell growth during fermentation.

#### 2.2.2. Microbial Synthesis of Beta-Alanine via Acetyl-CoA-FUM-L-Asp Pathway

Pyruvate is converted to acetyl-CoA by pyruvate dehydrogenase (PDH) and enters the TCA cycle. As indicated in Figure 3B, the TCA intermediate fumarate can be converted to L-Asp by aspartate ammonia lyase (AspA) [56], and ADC catalyzes the conversion of L-Asp to beta-alanine, realizing the synthesis of beta-alanine using fumarate, which is much less expensive than L-aspartate as a substrate. Usually, the fed-batch fermentation was carried out under the initial concentration of fumarate with 1500 mM at pH 7.0, and an agitation of 500 rpm at 37 °C. Fumarate can also be synthesized from maleic acid (via maleic acid isomerase), further expanding the availability of raw materials [19]. However, fumarate is typically derived from fossil resources, which is unsustainable, and enzyme purification for large-scale production remains costly [64].

Fatty acids (FAs) possess a higher degree of reduction and mass-based carbon content (75%) relative to glucose. As promising alternative feedstocks to glucose, FAs can be readily obtained from diverse sources, including waste oils, crude oils, and oil by-products [65]. An alternative pathway to supply the TCA cycle intermediates is through the glyoxylate shunt and the oxidative branch of the TCA cycle as shown in Figure 2. Song et al. reported beta-alanine production from glucose by coupling the glyoxylate shunt with the PEPC pathway to sustain L-Asp supply, achieving a titer of 32.3 g/L and an overall yield of 0.135 g/g glucose [66]. Miao et al. further demonstrated efficient beta-alanine biosynthesis, with a yield of 35.57 g/L in shake-flask cultivation using palmitic acid as the feedstock, and an overall yield of 72.05 g/L in fed-batch cultivation employing soybean oil (a lipid composition analogous to tripalmitin) as the carbon source [67]. The use of FA-based feedstocks enables high-yield beta-alanine production without disrupting TCA cycle flux, indicating that FAs represent an ideal carbon source for the biosynthesis of other TCA cycle-derived chemicals. Furthermore, FA feedstocks support higher ATP and NAD(P)H availability than glucose, implying that FAs are more advantageous for the biosynthesis of a broad spectrum of value-added metabolites.

#### 2.2.3. Microbial Synthesis of Beta-Alanine via 2-PGA-OAA-L-Asp Pathway

The conversion of waste glycerol into high-value C4 hydrocarbons represents an attractive and promising strategy for the valorization of waste renewable resources [68]. Previous studies have established a green biocatalytic route that enables the three-carbon compound glycerol to be converted into phosphoenolpyruvate (PEP), as illustrated by the pink arrow in Figure 2. Specifically, the glycerol is converted into glyceraldehyde by *Ardenticatenaceae bacterium* (*A. bacterium*) alditol oxidase (AbALDO) and then form glycerate (GA) by *E. coli* lactaldehyde dehydrogenase (EcALDH) with a specific activity of 4.23 U/mg^−1^ [69]. Glycerate then undergoes phosphorylation catalyzed by *Pyrococcus horikoshii* (*P. bacterium*) glycerate 2-kinase (GK) to generate 2-phosphoglycerate (2-PGA), which is subsequently dehydrated by *Carbox ydocella* (*C. ydocella*) phosphopyruvate hydratase (CyPPH) to yield PEP [69]. As detailed in Figure 2, the resulting PEP is then carboxylated to form OAA and L-Asp, the direct precursor for beta-alanine biosynthesis.

This pathway is divided into two modules: the glycerol utilization module realizes the conversion of glycerol to glycerate; the CO_2_ fixation module realizes the conversion of glycerate to L-Asp. As shown in Figure 2, the glycerol utilization module showcases non-natural pathways established via AbALDO for glycerol utilization, which are further refined based on the comprehensive pathway design. The initial step of glycerol involves the catalytic action of AbALDO to convert glycerol to glyceraldehyde and the by-product H_2_O_2_ [70]. Subsequently, the produced H_2_O_2_ is decomposed by *E. coli* CAT (EcCAT) into H_2_O and O_2_. The subsequent step in glycerol oxidation incorporates EcALDH to facilitate the conversion of glyceraldehyde to glycerate, alongside the conversion of the cofactor NAD^+^ to NADH [71]. This process provides the initial substrates and essential cofactor NADH required for subsequent CO_2_ fixation modules.

More importantly, rational modular pathway engineering enabled the simultaneous achievement of multiple critical goals: the introduction of EcALDH not only successfully drove the regeneration of the costly cofactor NADH, but also significantly accelerated glycerol utilization; ATP regeneration was realized via an ATP recycling system mediated by polyphosphate kinase (PPK); and efficient CO_2_ fixation was accomplished using the high-activity carbon-fixing enzyme PPC, which is a key enzyme in the plant C4 pathway derived from *Canabis sativa* (*C. sativa*) [72]. Under optimized reaction conditions, 8.9 mM GA was produced from glycerol within 2 h, and the subsequent two-module cascade reaction further converted GA into 35 mM L-Asp via the CO_2_ fixation module over the same 2.0 h timeframe [68]. However, the conversion rate of glycerol to glycerate remains relatively slow compared to other enzymatic steps, representing a persistent bottleneck for process optimization. Additionally, biological CO_2_ fixation poses inherent thermodynamic and efficiency limitations relative to chemical and electrochemical fixation strategies [73]. Recently, based on *E. coli* serving as the dominant engineered bacterial hosts for heterologous expression of core catalytic enzymes, owing to their rapid growth kinetics, high heterologous protein expression efficiency, and straightforward genetic manipulation, advanced strategies based on engineered artificial microbial whole-cell factories have been developed.

#### 2.2.4. Microbial Synthesis of Beta-Alanine via Glucose-OAA-FUM-L-Asp Pathway

*C. glutamicum* is a facultative aerobic, fast-growing, Gram-positive bacterium that can act as a cell factory utilizing a wide range of sugars, organic acids, and alcohols as either sole or mixed substrates [74]. Owing to its GRAS (Generally Recognized as Safe) status and robust metabolic plasticity, *C. glutamicum* has emerged as a model industrial microorganism and a promising chassis for constructing environmentally sustainable microbial cell factories [75].

**Figure 4:**
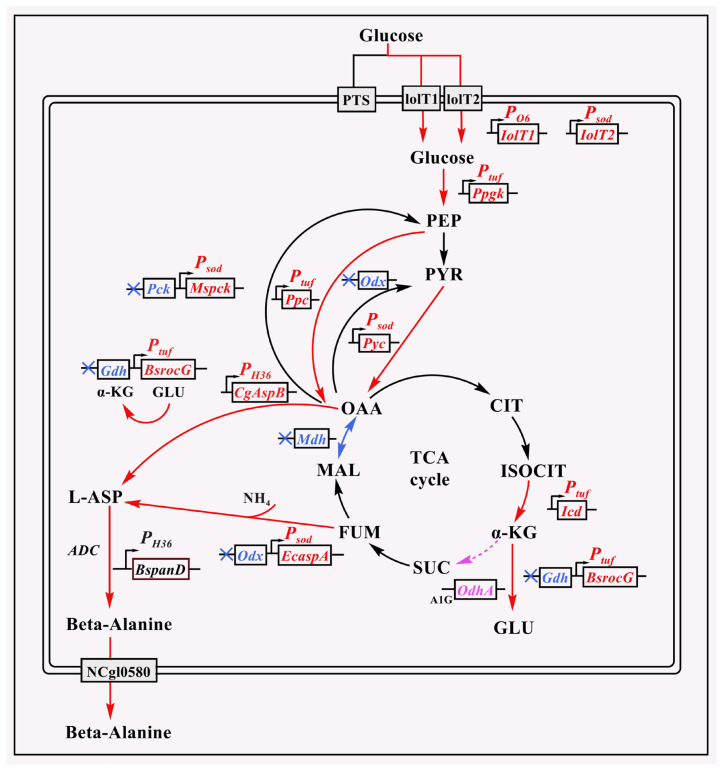
Overview of whole-cell catalysis production of beta-alanine in engineering conducted *C. glutamicum* The presented genes and their encoding proteins are: *EcAspA*, *E. coli* aspartate ammonia-lyase; *BsPanD*, *B. subtilis* aspartate 1-decarboxylase (ADC); *BsRocG*, *B. subtilis* glutamate dehydrogenase from; *CgAspB*: *C. glutamicum* aspartate aminotransferase; *MsPck*, *M. succiniciproducens* phosphoenolpyruvate carboxykinase; *Pck*, *C. glutamicum* original phosphoenolpyruvate carboxykinase; *Gdh*, *C. glutamicum* original glutamate dehydrogenase; *Icd*, isocitrate dehydrogenase; *IolT1*, myo-inositol permease 1; *IolT2*, myo-inositol permease 2; *Mdh*, malate dehydrogenase; *OdhA*, α-ketoglutarate dehydrogenase; *Odx*, *C. glutamicum* original oxaloacetate decarboxylase; *MsPck*, *M. succiniciproducens* phosphoenolpyruvate carboxykinase; *Ppc*, phosphoenolpyruvate carboxylase; *Ppgk*, glucokinase; *Pyc*, pyruvate carboxylase; Metabolite names are: PEP, phosphoenolpyruvate; PYR: pyruvate; OAA: oxaloacetate; CIT, citrate; ISOCIT, isocitrate; α-KG, α-ketoglutarate; SUC: succinate; FUM, fumarate; MAL, malate; GLU, L-glutamate; L-Asp: L-aspartate; PTS, phosphotransferase system.

Distinct from all above, Ghiffary et al. engineered *C. glutamicum* strain ATCC 13032 for high-efficiency beta-alanine biosynthesis through systematic metabolic engineering [56]. As illustrated in Figure 4, the research team first screened ADCs encoded by *PanD* genes from seven distinct bacterial strains, including *C. glutamicum* (*CgPanD*), *B. subtilis* (*BsPanD*), *E. coli* (*EcPanD*), *Rhodococcus opacus* (*RoPanD*), *Streptomyces griseus* (*SgPanD*), *Bacillus thuringiensis* (*BtPanD*), and *Saccharopolyspora erythraea* (*SePanD*), identifying the ADC from *B. subtilis* as the most catalytically efficient for beta-alanine synthesis. Second, the native phosphotransferase system (PTS) for glucose uptake consumes PEP during sugar import [76], which reduces the availability of PEP for synthesizing beta-alanine precursors (OAA and L-Asp) and severely limits product titers. To mitigate this drawback, Ghiffary et al. optimized a PTS-independent glucose uptake pathway, comprising the overexpression of myo-inositol permease 1 (*IolT1*), myo-inositol permease 2 (*IolT2*), and glucokinase (*Ppgk*), to preserve intracellular PEP pools. Third, they strengthened key genes governing precursor accumulation to boost metabolic flux toward beta-alanine, including *Ppc* (encoding PEP carboxylase for PEP-to-OAA conversion), *Pyc* (encoding pyruvate carboxylase for pyruvate-to-OAA conversion), *Icd* (encoding isocitrate dehydrogenase for isocitrate-to-α-KG conversion), and *AspA* (encoding aspartate ammonia-lyase for FUM-to-L-Asp conversion). Fourth, the identification and overexpression of a putative beta-alanine exporter (Ncgl0580) significantly enhanced the extracellular accumulation of beta-alanine production [56]. Finally, by fed-batch fermentation using a 5 L bioreactor with pH 7.0, the dissolved oxygen (DO) level at 30% (*v*/*v*) and agitation speed between 600 and 1000 rpm, the final engineered *C. glutamicum* strain yielded 166.6 g/L beta-alanine, with a corresponding yield of 0.28 g/g glucose and productivity of 1.74 g/L/h [56].

In order to objectively compare strategies for beta-alanine production and identify trends, we listed the representative research to summarize the final titer (g/L), the host organism, carbon source, etc., as shown in Table 2. In summary, comparative analysis of current beta-alanine biosynthesis strategies—including enzyme cascade-mediated biotransformation and microbial production via whole-cell catalysis—reveals that the metabolically engineered *C. glutamicum* strain exhibits the optimal production performance (Table 2). Notably, this strain can efficiently secrete beta-alanine into the extracellular matrix, greatly simplifying downstream separation and purification processes. To the aspect of beta-alanine whole-cell biosynthesis with potential industrial application, the producing industrial strains majorly focused on engineered bacteria; there was no report using industrial *S. cerevisiae* and filamentous fungi, although many yeasts have been found to de novo synthesize beta-alanine and its derivatives [3,77,78].

However, there are two pivotal factors for the microbial production of beta-alanine that are worthy of note. First, from the considerations of techno-economic evaluation, regardless of which routine is chosen for beta-alanine biosynthesis via whole-cell biocatalysis, ADC (encoded by *panD*) is the final and rate-limiting enzyme in virtually all reviewed biocatalysis strategies [79]. There are two main types of ADC that have been isolated and used in previous studies. The first type of ADC exists in bacteria in the homotetramer state, with a pyruvyl group as a cofactor [80]. The other type of ADC is mainly isolated from insects and exists as a homodimer with pyridoxal-5’-phosphate (PLP) as a cofactor [81,82]. The amino acid sequence and three-dimensional structure of ADCs from bacteria and insects are quite different, indicating that they evolved independently. ADCs from bacteria including *B. subtilis*, *E. coli* and *C. glutamicum* have been well studied and used for beta-alanine synthesis in the laboratory [61,81]. Nevertheless, ADCs derived from bacteria show substrate-dependent inactivation. Bacterial ADCs perform autocatalytic intramolecular self-cleaving reactions on conserved serine residues and generate a pyruvyl moiety, which is essential for their catalytic activity in the L-ASP decarboxylation reaction [83]. For instance, the ADC from *E. coli* (also known as the *PanD*) is initially synthesized as a pro-enzyme (π-Chain, 13.8 kDa), and then it is subjected to self-shear processing at Gly24-Ser25 to generate an 11 kDa beta-chain with a hydroxyl group at the C-terminus and a 2.8 kDa α-chain with a pyruvyl group at the N-terminus. It has been observed that the abnormal protonation of the imine structure during the L-ASP decarboxylation reaction results in irreversible modification of the active site and loss of the catalytic function. However, the structure and catalytic mechanism of insect ADCs are different from those of bacterial ADCs, with no mechanism-based inactivation effect, which enables them to become an excellent candidate for beta-alanine synthesis. Therefore, large amounts of the bacterial ADC have been invested into the reaction for the sake of replacing the inactivated ADC during the bioconversion process, which is of low efficiency and high cost [38,83]. So far, several insect ADCs including those from *Aedes aegypti* (*Ae*ADC), *Drosophila melanogaster* (*Dm*ADC), and *Tribolium castaneum* (*Tc*ADC) have been investigated, yet only the *Tc*ADC has been employed in the synthesis of beta-alanine [84,85,86]. In 2023, Liu et al. characterized an *Mp*ADC from an aphid, *Myzus persicae*. It showed excellent catalytic activity at pH 6.0–7.5 and 37 °C. With the help of chaperone co-expression and N-terminal engineering guided by AlphaFold2 structure prediction, the expression and catalytic ability of *Mp*ADC in *E. coli* were significantly improved. Using 50 g/L of *E. coli* cells expressing an *Mp*ADC variant cultured in a 15 L fermenter, 232.36 g/L of beta-alanine was synthesized in 13.5 h, with the average beta-alanine yield of 17.22 g/L/h, which is the best known productivity so far [38].

Second, the beta-alanine accumulation at gram-per-liter concentrations exerts demonstrable osmotic stress and metabolic effects on bacterial hosts in methodology logic although a few studies discuss this point. In our viewpoint, beta-alanine owns a characterized feature of dramatically high water solubility of 545 g/L at 25 °C [87], implicating the great tendency for high preservation in cell cytoplasm instead of being excreted, which constructs major challenges that scientists would address by developing biosynthesis. To avoid substrate inhibition and high osmotic pressure, and reduce the accumulation of toxic by-products, some research has alleviated the possible toxicity and feedback inhibition by beta-alanine via fed-batch fermentation [58,67]. For instance, the fed-batch fermentation of the final engineered *C. glutamicum* strain yielded the highest 166.6 g/L beta-alanine until now [56]. In the future, a series of methodologies could be considered, including screening the appropriate exporter protein for the synthesized beta-alanine transport to the extracellular space for further isolation, or inactivating the beta-alanine-utilizing enzymes, for example, 4-aminobutyrate aminotransferase (ABAT), which is a key pyridoxal phosphate (PLP)-dependent catabolic enzyme catalyzing the reversible transamination of γ-aminobutyric acid (GABA) and beta-alanine with α-ketoglutarate, converting GABA into semialdehyde and generating L-glutamate simultaneously, by which the yield of beta-alanine could be efficiently maintained [88].

## 3. Perspectives

Recent advances have delineated multiple biosynthetic strategies for beta-alanine using diverse key precursors and catalytic enzymes. Despite these breakthroughs, substantial challenges persist owing to the inherent complexity of each biosynthetic pathway.

First, nearly all characterized enzymes involved in beta-alanine biosynthesis suffer from suboptimal catalytic activity and efficiency, as well as partial substrate promiscuity that triggers byproduct formation, ultimately hindering downstream product synthesis. As a promising solution, AI (artificial intelligence)-driven iterative protein engineering has emerged as a powerful tool to accelerate the directed evolution of high-performance enzyme variants via design-build-test-learn (DBTL) cycles. Even if the DBTL cycle has not been fully applied specifically to beta-alanine yet, there are already examples where these approaches have been used for enzymatic and microbial synthesis of other compounds. For example, Pandi et al. described METIS (Machine-learning guided Experimental Trials for Improvement of Systems, named after the ancient goddess of wisdom and crafts, Μῆτις, *lit*. “wise counsel”), a modular and versatile active machine learning workflow for data-driven optimization of a biological objective function (an output/target that depends on multiple factors) with minimal datasets [89]. In this research, they built a workflow for improving these systems between one and two orders of magnitude. These applications included cell-free transcription and translation, genetic circuits, and a 27-variable synthetic CO_2_ fixation CETCH (crotonyl-CoA/ethylmalonyl-CoA/hydroxybutyryl-CoA) cycle, which is also called a new-to-nature synthetic CO2-fixation cycle. This methodology opens a novel way for convenient optimization and prototyping of genetic and metabolic networks with customizable adjustments according to user experience, experimental setup, and laboratory facilities.

Second, the intricate nature of microbial metabolic networks often leads to metabolic imbalance, toxic intermediate accumulation, and carbon flux diversion toward competing pathways, limiting beta-alanine titers and yields [90]. For example, Liu et al. employed Alphafold2-based biological information analysis to screen and predict the structure of ADC, and then by a combined verification between the ADC molecular modification-based model and experimental screening process, the optimal ADC with high activity and stability was figured out [38]. Meanwhile, the authors also took a relatively long time on the first “method” exploration on the “IT (information technology)” and “BT (biotechnology)” based strategy, providing a very good reference for future whole-cell catalysis strategy screening.

**Figure 5:**
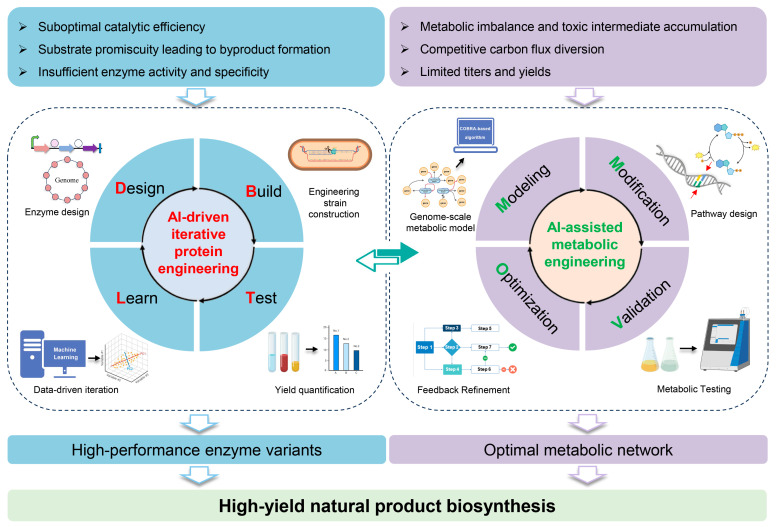
Schematic diagram of AI-enabled dual-strategy engineering for high-yield natural product biosynthesis. In AI-driven iterative enzyme engineering, the design-build-test-learn (DBTL) cycle initiates from enzyme design, proceeds to engineered strain construction, yield quantification, and data-driven iteration, ultimately enabling the identification of high-performance enzyme variants. In AI-assisted metabolic engineering, genome-scale metabolic models integrated with COBRA-based algorithms guide metabolic pathway design and modification, followed by experimental validation and feedback optimization to establish an optimal metabolic network.

In the future, as shown in Figure 5, first, leveraging existing datasets will encompass beta-alanine-related enzyme sequences, three-dimensional structures, and functional phenotypes. Machine learning (ML) models will predict high-potential mutation sites—such as flexible loops near the active pocket, residues governing surface charge distribution, or amino acids mediating intermolecular interactions—and guide the construction of focused mutant libraries. These targeted libraries are far smaller than those generated via random mutagenesis, drastically reducing experimental workload and accelerating enzyme optimization. Although machine learning (ML) has not yet been extensively applied to beta-alanine biosynthesis, this approach has been successfully utilized to optimize two novel glycyl-CoA carboxylase (GCC) M5 variants [91]. Derived from propionyl-CoA carboxylase (PCC) via five-site mutagenesis, GCC M5 serves as an unnatural enzyme that catalyzes the pivotal pathway reaction and enhances photosynthetic carbon dioxide fixation [92]. These optimized variants exhibit a twofold higher carboxylation rate and a 60% reduction in energy consumption, effectively overcoming kinetic and thermodynamic constraints of the tartronyl-CoA (TaCo) pathway. ML-assisted workflows substantially cut down the workload for high-performance enzyme screening, demonstrating great prospects of integrating machine learning with directed evolution in enzyme engineering [91].

Second, AI-assisted metabolic engineering has shown great promise (Figure 5): the integration of genome-scale metabolic models (GEMs) with COBRA-based algorithms will enable rational design of optimal metabolic pathways, maximizing target flux toward high-value products (e.g., paclitaxel) in whole-cell factories while minimizing cellular metabolic burden [93].

In summary, the integration of AI with enzyme engineering and metabolic engineering holds transformative potential for revolutionizing industrial production of bioactive substances like beta-alanine. The development of hybrid AI systems capable of synchronously optimizing enzyme kinetics and global metabolic flux, coupled with the construction of standardized biochemical databases for metabolism-related pathways, will not only accelerate the development of super-performing enzyme screening with high activity and conditional stability, but also provide a universal blueprint for the biosynthesis and industrial scale-up of whole-cell biocatalysis of other high-value natural products.

## 4. Conclusions

Beta-alanine holds great market potential for extensive applications in the chemical, pharmaceutical and food industries, yet its large-scale industrial production still faces considerable challenges. This review systematically summarizes current biosynthesis strategies for beta-alanine, covering in vitro cell-free enzymatic catalysis and in vivo microbial whole-cell catalysis. Special focus is placed on enzyme cascade systems, metabolic engineering modifications and industrially oriented biosynthetic pathways. To date, the production of beta-alanine remains largely at the laboratory stage. Comparative analysis of product yield and metabolic pathway-associated production costs indicates that microbial whole-cell synthesis might exhibit superior performance and broader application prospects relative to in vitro enzymatic catalysis from our viewpoint. Advances in AI-driven DBTL cycles and machine learning are promising to revolutionize mechanistic studies of beta-alanine biosynthesis and accelerate the realization of large-scale industrial manufacturing. Overall, this review deepens the understanding of key enzymes and their catalytic metabolic reactions, which constitute the core components of integrated biosynthetic systems. The insights presented here also lay a solid foundation and point the direction for the future industrial biosynthesis of beta-alanine.

## Figures and Tables

**Table 1:** Enzyme-catalytic biosynthesis of beta-alanine from different substrates.

Substrate	Enzymes	Intermediate	Final Titer (g/L)	Reference
1,3-diaminopropane	ApDAO, AcAPALDH	3-aminopropionaldehyde	26.8 mg/L/h	[18]
L-asparate	CgPanD	-	35.69 mg/L/h	[33]
Fumarate	EcMAL, CgPanD	L-asparate	80% theoretical yield within 2 h	[32]
3-aminopropionitrile	Nitrilase BjNIT3397	3-aminopropanamide	n/a	[34]

**Table 2:** Whole-cell synthesis of beta-alanine by different strategies.

Host Organism	Carbon Source	Intermediate	Enzymes	Final Titer (g/L)	Reference
*E. coli*	Glucose	L-Asparate	PanD, AspA, Acs	0.83 g/L/h	[58]
*E. coli*	Glucose	L-Asparate	PanD, AspC	1.58 g/L/h	[59]
*E. coli*	Glucose	L-Asparate	PanD, AspB	1.05 g/L/h	[60]
*C. glutamicum*	Glucose	L-Asparate	PanD	0.79 g/L/h	[61]
*E. coli*	Glucose	Oxaloacetate	AspB, *ppc*	1.0 g/L/h	[62]
*E. coli*	Glucose	Fumarate	PanD, AspA	0.62 g/L/h	[62]
*E. coli*	Fatty acid	Acetyl-CoA	PanD, AspA	1.47 g/L/h	[67]
*E. coli*	Glycerol	2-Phosphoglycerate, Phosphoenolpyruvate	ALDO, ALDH	-	[73]
*C. glutamicum*	Glucose	All intermediates involved in glycolysis and TCA	Systematic metabolic engineering	1.74 g/L/h	[56]
*E. coli*	Glucose	L-Asparate	PanD	17.22 g/L/h	[38]

## Data Availability

Data sharing is not applicable. No new data were created or analyzed in this study.
